# Supplementary material for: Differential effects of intra-modal and cross-modal reward value on perception: ERP evidence
Source: PLoS One. 2023 Jun 30;18(6):e0287900. doi: 10.1371/journal.pone.0287900 (PMC10313067; doi:10.1371/journal.pone.0287900)
Supplement: S1 Text — (DOCX) [file pone.0287900.s001.docx]

# S1 Text

## ERP results when contralateral posterior ERPs were examined

We evaluated the amplitude of ERP components (PA, P1, N1, and P3) in contralateral electrodes (**S5 Figure**). To this end, responses of O2 and PO8 to the stimuli presented on the left hemi-field and responses of O1 and PO7 to the stimuli on the right hemi-field were measured. The contralateral ERPs were then averaged across the two sides. Two-way ANOVAs with reward value and modality as independent factors and amplitude of each component as dependent factor revealed only a trend for an interaction between reward value and modality in N1 window (F_(1,35)_ = 3.19, p = 0.083, η_p_^2^ = 0.083), corresponding to a decrease in N1 negativity for cross-modal, high- compared to low-value condition (mean ± s.e.m: 0.41 ± 0.28 and -0.49 ± 0.39 for high- and low-value stimuli respectively, t(35) = 2.3, p = 0.025, d_z_ = 0.389). Furthermore, a main effect of reward value was found in P3 window (F_(1,35)_ = 9.14, p = 0.005, η_p_^2^ = 0.207). Planed pairwise comparisons revealed a significant enhancement of contralateral P3 responses for high- compared to low-value cross-modal cues (mean ± s.e.m: 0.5 ± 0.28 and -0.59 ± 0.44, for high- compared to low-value cross-modal cues respectively, t(35) = 2.388, p = 0.022, d_z_ = 0.398).

## P3 responses of midline electrodes during conditioning

Previous studies have reported robust reward modulations of P3 responses of midline electrodes (Pz, CPz, Cz, FCz and Fz) time-locked to the presentation of reward cues, both for both visual and auditory cues [1–5]. Based on these and our pre-registered plan, we next inspected the ERP responses of the midline electrodes (**S1 Figure**), time-locked to the presentation of the reward cues in the P3 window (300-600 ms). The frontocentral electrodes are most sensitive to auditory signals (Fz, FCz), whereas the posterior midline electrodes are most sensitive to visual cues (Cz, Pz) or both (Cz), based on the studies reported above. Therefore, we expected to find a significant difference in P3 amplitudes for stimuli of different modalities, reflecting the different topography of areas processing visual or auditory information. In addition, we also expected an effect of reward value for one or both modalities across these electrodes. We did not find an overall robust value-driven modulation based on our preregistered analysis plan. Specifically, a rmANOVA revealed a main effect of modality (F(1,35) = 4.64, P = 0.038, ηp2= 0.117), a main effect of electrode (F(4,140) = 124.73, p < 0.001, ηp2= 0.781) and a trend for modality and electrode interaction (F(4,140) = 3.53, p = 0.054, ηp2= 0.092), as expected. Other comparisons did not reach significance: main effect of value (F(1,35) = 1.6, p = 0.214), interaction between value and modality (F(1,35) = 3.19, p = 0.083), interaction between value and electrode F(1,35) < 1, p = 0.381) and interaction between factors value, modality and electrode (F(1,35) = 1.34, p = 0.054). Although the inspection of ERP responses (**S1 Figure**) suggests value-driven modulations in some of these electrodes for visual (esp. Pz), auditory (esp. Fz), or both cues types (Cz), in order to adhere to our preregistered plan, we did not further investigate these effects.

## ERP results when only correct trials were included

In our pre-registered analysis plan, we had intended to compare the ERP effects of the correct and error trials. However, after the data acquisition, we noticed that we could not have a noise-free estimation of the ERPs of error trials, as in some cases errors were too infrequent (Mean ± SD: 6.6 ± 2.4 and 13.6 ± 5.4, for number of error trials in pre- and post-conditioning, respectively). Therefore, we did not undertake this pre-registered analysis, albeit we tested whether we obtain similar results when we only include correct trials in our analysis (for which we had sufficient number of trials; i.e.,> 25 trials for each condition and each phase). To this end, we examined the ERP responses of the posterior ROI when only correct trials of each condition were included in our analysis (**S6 Figure**). To this end, we performed rmANOVAs on the amplitude of PA, P1, N1 and P3 and the latency of P1 and N1 components of the ERPs with reward value (high or low) and modality (intra-modal or cross-modal) as factors. Overall, we obtained the same results as results obtained with all trials, albeit some of our reported were even stronger when only correct trials were included.

In PA (90-120 ms) window we found an interaction effect between factors modality and reward value (F(1,35) = 10.54, p = 0.003, ηp2 = 0.231). Intra-modal high-value cues suppressed PA amplitudes compared to low-value cues (mean ± s.e.m: -0.58 ± 0.35 and 0.41 ± 0.27, for the PA amplitude of high- compared to low-value cues, t(35) = -2.475, p = 0.018, dz = 0.412). Cross-modal high value cues on the other hand increased the PA amplitude compared to low value cues (mean ± s.e.m: 0.02 ± 0.2 and -0.86 ± 0.33, for the PA amplitude of high- compared to low-value cues, t(35) = 2.278, p = 0.029, dz = 0.380).

Analysis of P1 component revealed only a main effect of modality (F(1,35) = 5.33, P = 0.027, ηp2 = 0.132) but no main or interaction effect with reward value (both ps>0.1).

In N1 (170-250 ms) window, we found an interaction effect between reward value and modality (F(1,35) = 6.46, P = 0.016, ηp2 = 0.156). Cross-modal high-value cues decreased the N1 negativity compared to low value cues (mean ± s.e.m: 1.21 ± 0.37 and -0.46 ± 0.41, t(35) = 3.245, p = 0.003, dz = 0.541). The modulation of N1 amplitude was not significant for intra-modal cues.

In P3 window (300-600 ms), we found no main effect of modality, reward value or an interaction between reward and modality.

The analysis of the latency of P1 and N1 components revealed only a significant value × modality interaction effect for N1 latency (F(1,35) = 6.38, P = 0.016, ηp2 = 0.154). Intra-modal, high value cues elicited faster N1 responses compared to low values cues (mean ± s.e.m: -4.94 ± 4.61 and 8.33 ± 3.39, for N1 latency of high- and low-value cues respectively corrected for pre-conditioning differences, t(35) = -2.228, p = 0.032, dz = 0.371).

## Analysis of pupil size during post-conditioing

As per our pre-registered plan, pupil size was measured as the percent signal change relative to the baseline (500 ms prior to the stimulus onset) in a time window from the stimulus onset until the response time. Similar to the analysis employed for the behavioral and ERP data, pupil size corresponding to each condition was corrected by subtracting the pre-conditioning data (see the Supplementary Information).To test the effect of reward value of intra- and cross-modal stimuli on pupil size, we performed a two-way ANOVA with factors value (high- low value) and modality (intra- and cross-modal). This analysis revealed no main effect of value (F(1,35) = 3.24, P = 0.080, ηp2 = 0.085), modality (F(1,35) = 0.03, P = 0.874, ηp2 = 0.001) or their interaction (F(1,35) = 0.05, P = 0.823, ηp2 = 0.001), hence we did not further investigate the correlation of pupil size effects with behavioral and ERP data as planned during the pre-registration.

## Analysis of eye position during post-conditioing

To ensure that our effects do not originate from changes in eye position due to reward value or modality, we conduct a two-way ANOVA with value (high- and low-value) and modality (intra- and cross-modal ) as independent factors and eye position relative to the fixation point during the presentation of the target as dependent factor. This analysis did not reveal any significant effect of value (F(1,35) = 1.44, P = 0.238, ηp2 = 0.039), modality (F(1,35) = 0.26, P = 0.614, ηp2 = 0.007) or their interaction (F(1,35) = 0.05, P = 0.816, ηp2 = 0.002). Finally, we also tested whether any of our previously reported reward effects on visual sensitivity (d’) and ERP components (P1, N1, and P300) was correlated with the eye position. This analysis did not show any significant correlation between changes in eye position and our reported effects. None of the correlational analyses showed an interaction between eye movements and previously reported reward effects. Therefore, these analyses rule out that our effects were driven by changes in eye position due to our factors of interest (reward or modality).

## Analysis of inter-individual differences in reward sensitivity

Based on our pre-registered plan, we tested whether participants' scores on BAS reward sensitivity test (as in Behavioral Inhibition System/Behavioral Approach System (BIS/BAS) Strobel, Beauducel, Debener, & Brocke, 2001) is correlated with our previously reported effects (average effect size of reward value across intra- and cross-modal stimuli during post-conditioing and corrected for pre-conditioing data). This analysis revealed no correlations of the BAS reward sensitivity scale and visual sensitivity (d’), amplitude (P1, N1, P300), and latency (P1, N1) of ERP components.

## Correlation of behavioral and ERP effects

We wondered whether the lack of a correlation of reward effects on ERPs with reward effects on behavior is due to averaging the responses of contralateral and ipsilateral electrodes in our ROI. Due to the strong retinotopic organization of early visual areas, the reward modulations of lateralized stimulus are strongest and most reliably correlated with behavior on the contralateral visual areas [6]. To this end, we performed an exploratory analysis where the responses of the contralateral electrodes of the posterior ROI measured in each time window (corresponding to PA, N1, and P3) were correlated with the behavioral d' of the corresponding condition (**S7 Figure**). This analysis revealed a significant positive correlation between value-driven modulations of the contralateral ERPs in N1 (r = 0.347, p = 0.037, Pearson Correlation, d_z_ = 0.74) and P3 (r = 0.352, p = 0.035, Pearson Correlation, d_z_ = 0.75) windows and behavioral d-primes of the cross-modal stimuli. These results hence indicate that the cross-modal reward effects on behavior may indeed rely on the value-driven response modulations of the contralateral visual areas. However, we note that these results should be treated with caution, as they reflect results obtained from an exploratory analysis and were not corrected for multiple comparisons.

## Citations

1. Krugliakova E, Klucharev V, Fedele T, Gorin A, Kuznetsova A, Shestakova A. Correlation of cue-locked FRN and feedback-locked FRN in the auditory monetary incentive delay task. Exp Brain Res. 2018;236: 141–151. doi:10.1007/s00221-017-5113-2

2. Gehring WJ, Willoughby AR. The medial frontal cortex and the rapid processing of monetary gains and losses. Science (80- ). 2002;295: 2279–2282. doi:10.1126/science.1066893

3. Krugliakova E, Gorin A, Fedele T, Shtyrov Y, Moiseeva V, Klucharev V, et al. The Monetary Incentive Delay (MID) Task Induces Changes in Sensory Processing: ERP Evidence. Front Hum Neurosci. 2019;13. doi:10.3389/fnhum.2019.00382

4. Glazer JE, Kelley NJ, Pornpattananangkul N, Mittal VA, Nusslock R. Beyond the FRN: Broadening the time-course of EEG and ERP components implicated in reward processing. International Journal of Psychophysiology. Elsevier B.V.; 2018. pp. 184–202. doi:10.1016/j.ijpsycho.2018.02.002

5. Van Den Berg B, Krebs RM, Lorist MM, Woldorff MG. Utilization of reward-prospect enhances preparatory attention and reduces stimulus conflict. Cogn Affect Behav Neurosci. 2014;14: 561–577. doi:10.3758/s13415-014-0281-z

6. Pooresmaeili A, FitzGerald THB, Bach DR, Toelch U, Ostendorf F, Dolan RJ. Cross-modal effects of value on perceptual acuity and stimulus encoding. Proc Natl Acad Sci U S A. 2014;111: 15244–15249. doi:10.1073/pnas.1408873111
