# Supplementary material for: Differential effects of intra-modal and cross-modal reward value on perception: ERP evidence
Source: PLoS One. 2023 Jun 30;18(6):e0287900. doi: 10.1371/journal.pone.0287900 (PMC10313067; doi:10.1371/journal.pone.0287900)
Supplement: S1 Fig — (DOCX) [file pone.0287900.s002.docx]

**
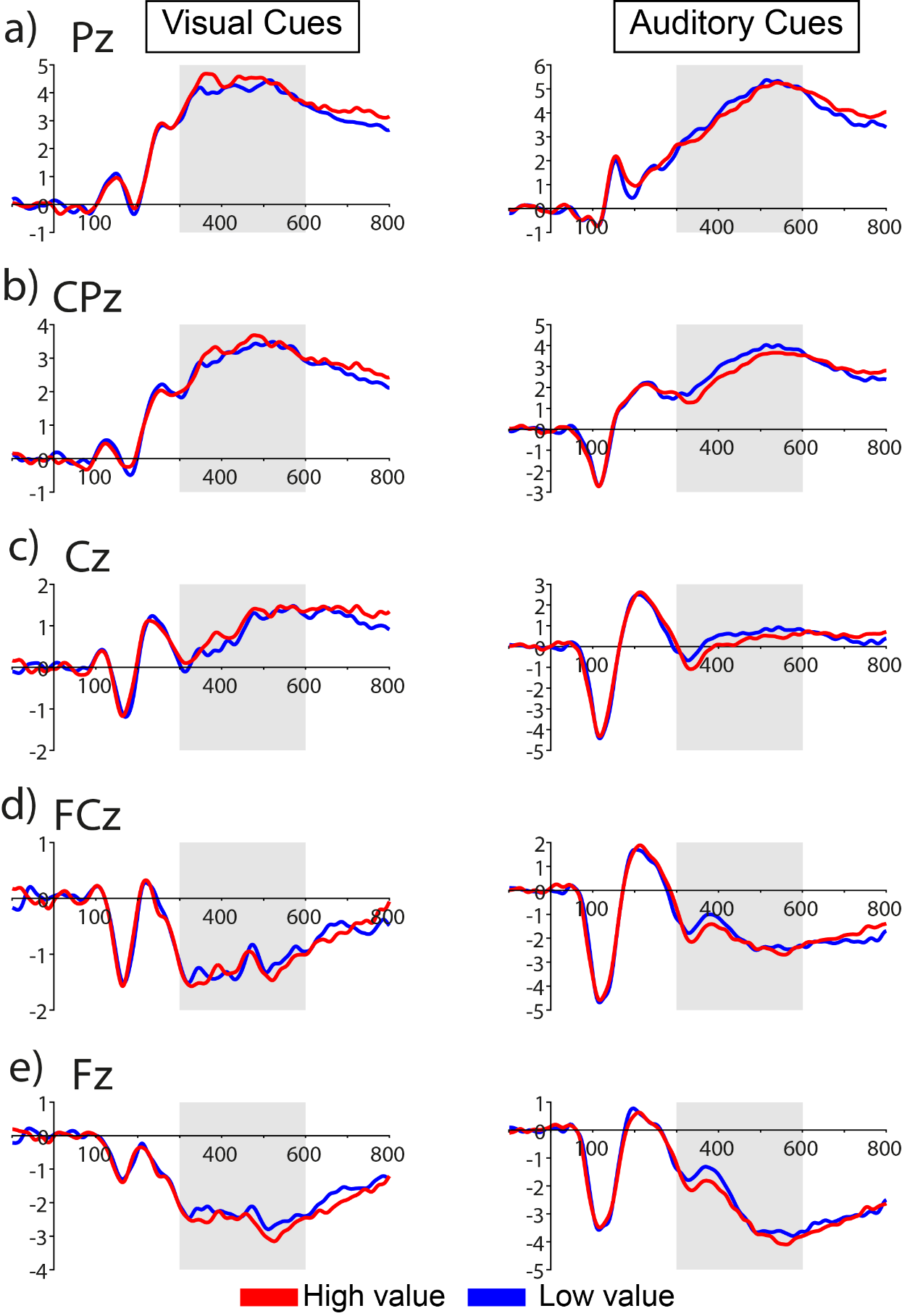
**

**S1 Figure. ERPs of midline electrodes during the reward associative learning (conditioning phase).**
