# Supplementary figures and images for: Differential effects of intra-modal and cross-modal reward value on perception: ERP evidence
Source: PLoS One. 2023 Jun 30;18(6):e0287900. doi: 10.1371/journal.pone.0287900 (PMC10313067; doi:10.1371/journal.pone.0287900)

**
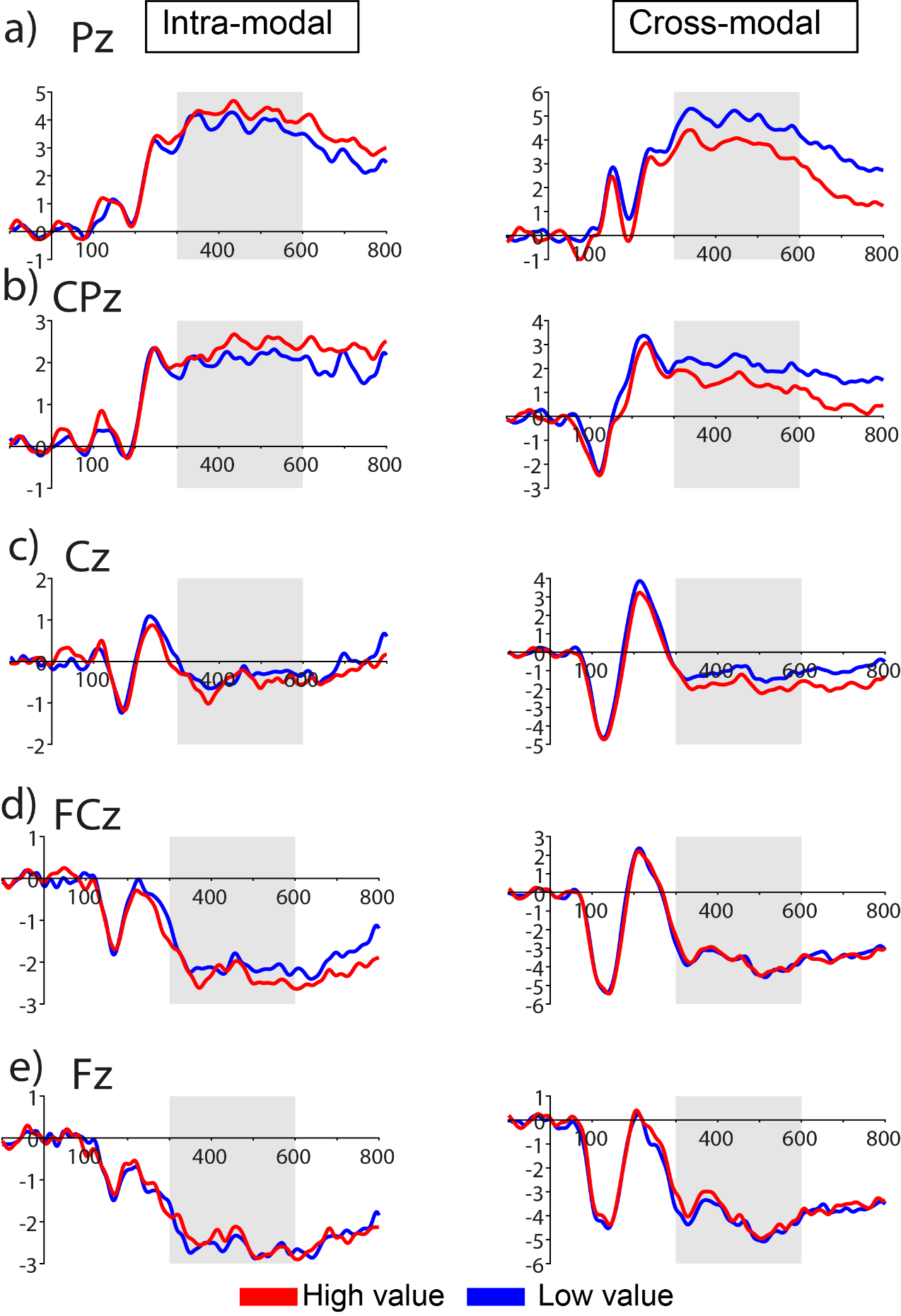
**

**S2 Figure. ERPs of midline electrodes during the pre-conditioning phase.**

Supplement: S2 Fig — (DOCX) [file pone.0287900.s003.docx]

**
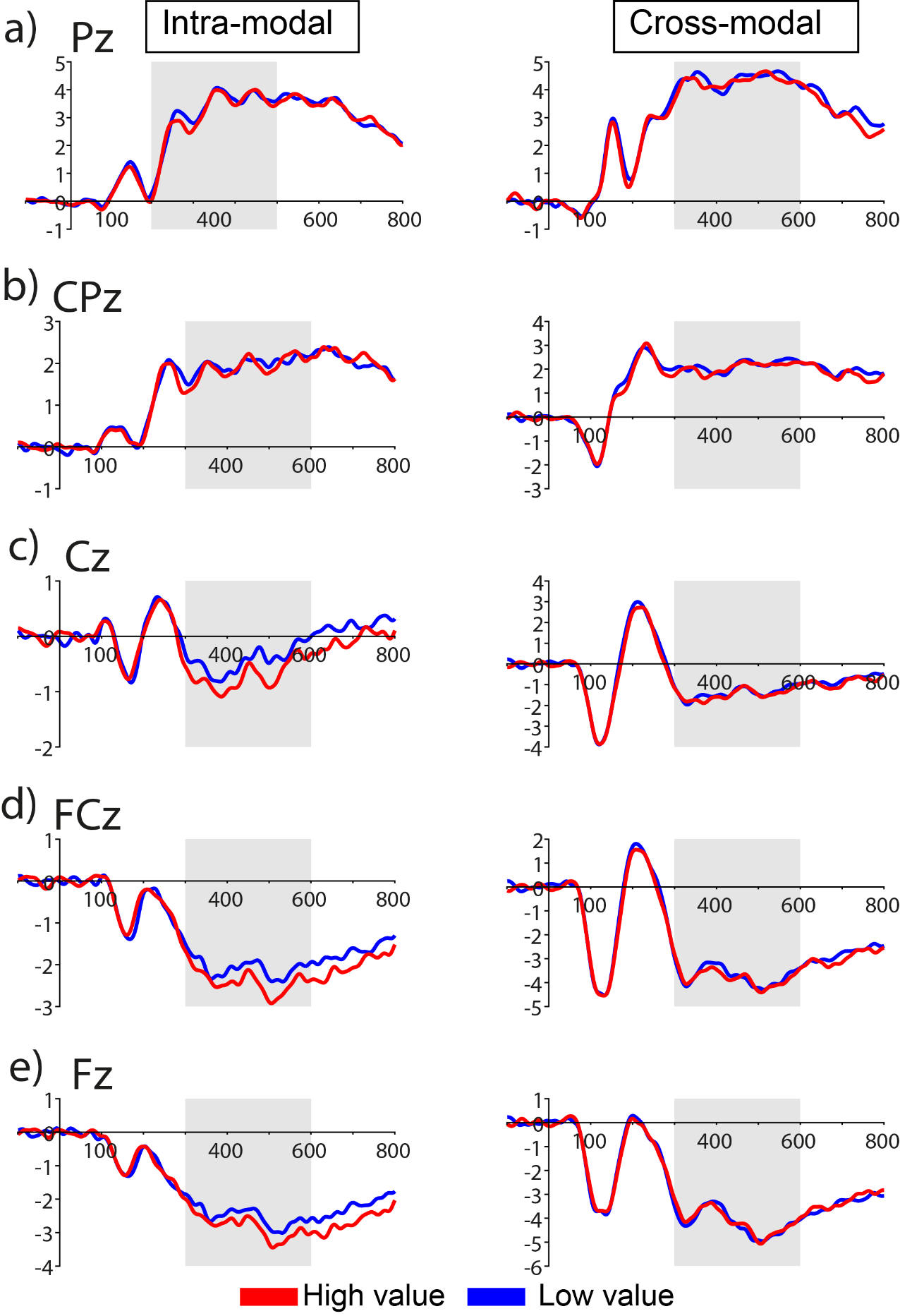
**

**S3 Figure . ERPs of midline electrodes during the post-conditioning phase.**

Supplement: S3 Fig — (DOCX) [file pone.0287900.s004.docx]
