# Supplementary material for: Differential effects of intra-modal and cross-modal reward value on perception: ERP evidence
Source: PLoS One. 2023 Jun 30;18(6):e0287900. doi: 10.1371/journal.pone.0287900 (PMC10313067; doi:10.1371/journal.pone.0287900)
Supplement: S4 Fig — (DOCX) [file pone.0287900.s005.docx]

**
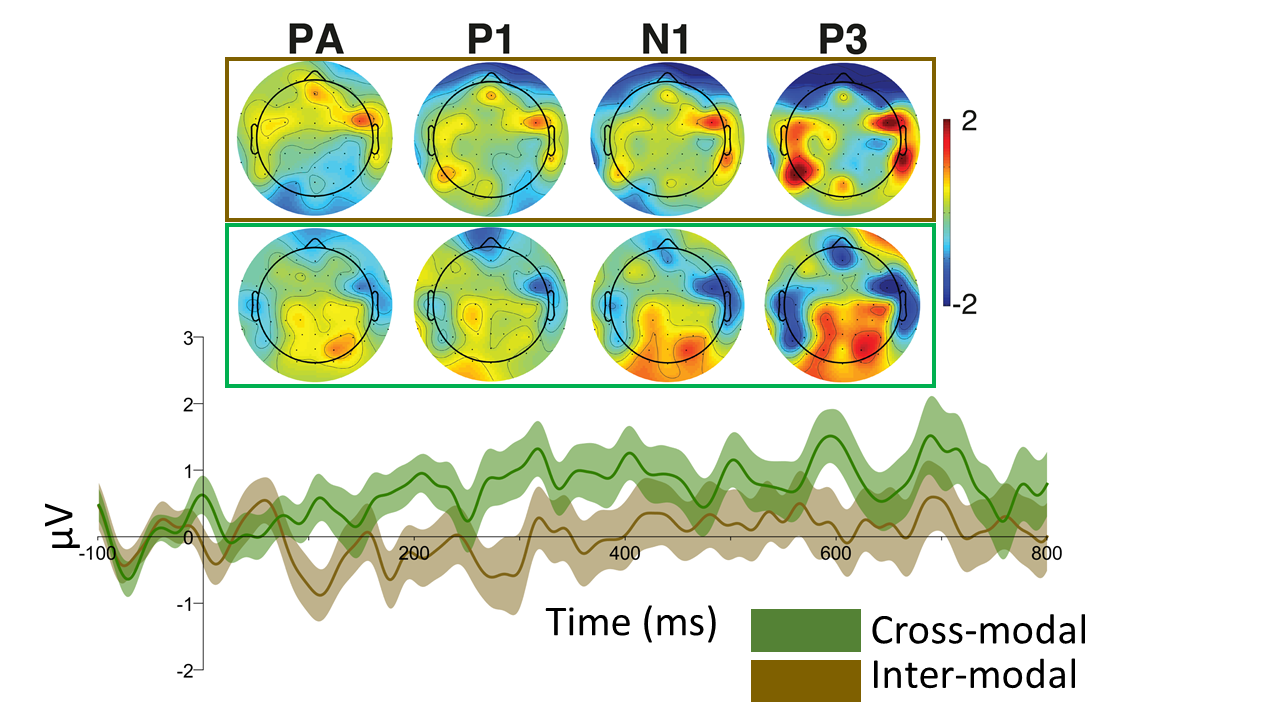
**

**S4 Figure. This figure should be compared to Figure 4 in the main text. Difference waves are shown for the cross-modal and inter-modal rewards, where each trace represents the effect of reward (high – low) in post-conditioing minus the same effect in pre-conditioing (see Figure 4b and 4c in the main text). The topoplots illustrate the topographic distribution of the reward effect in each time window for each condition (brown: inter-modal correspoidning to IH-IL and green: cross-modal corresponding to CH-CL).**
