# Supplementary material for: Differential effects of intra-modal and cross-modal reward value on perception: ERP evidence
Source: PLoS One. 2023 Jun 30;18(6):e0287900. doi: 10.1371/journal.pone.0287900 (PMC10313067; doi:10.1371/journal.pone.0287900)
Supplement: S5 Fig — (DOCX) [file pone.0287900.s006.docx]

**
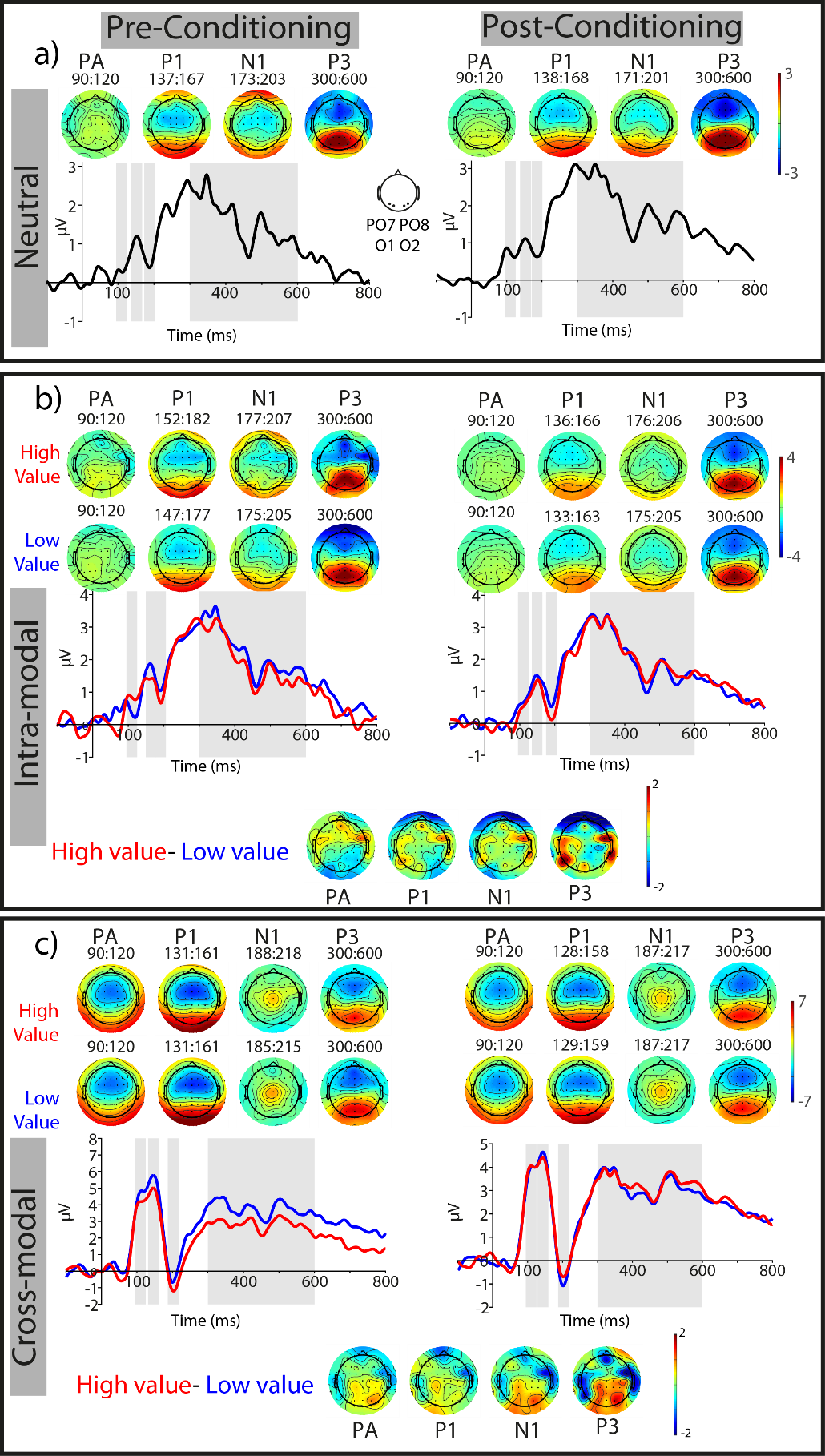
**

**S5 Figure. Contralateral responses of the posterior ROI during pre- and post-conditioning phases, see also Figure 4 in the main text.**
