# Supplementary material for: Differential effects of intra-modal and cross-modal reward value on perception: ERP evidence
Source: PLoS One. 2023 Jun 30;18(6):e0287900. doi: 10.1371/journal.pone.0287900 (PMC10313067; doi:10.1371/journal.pone.0287900)
Supplement: S6 Fig — (DOCX) [file pone.0287900.s007.docx]

**
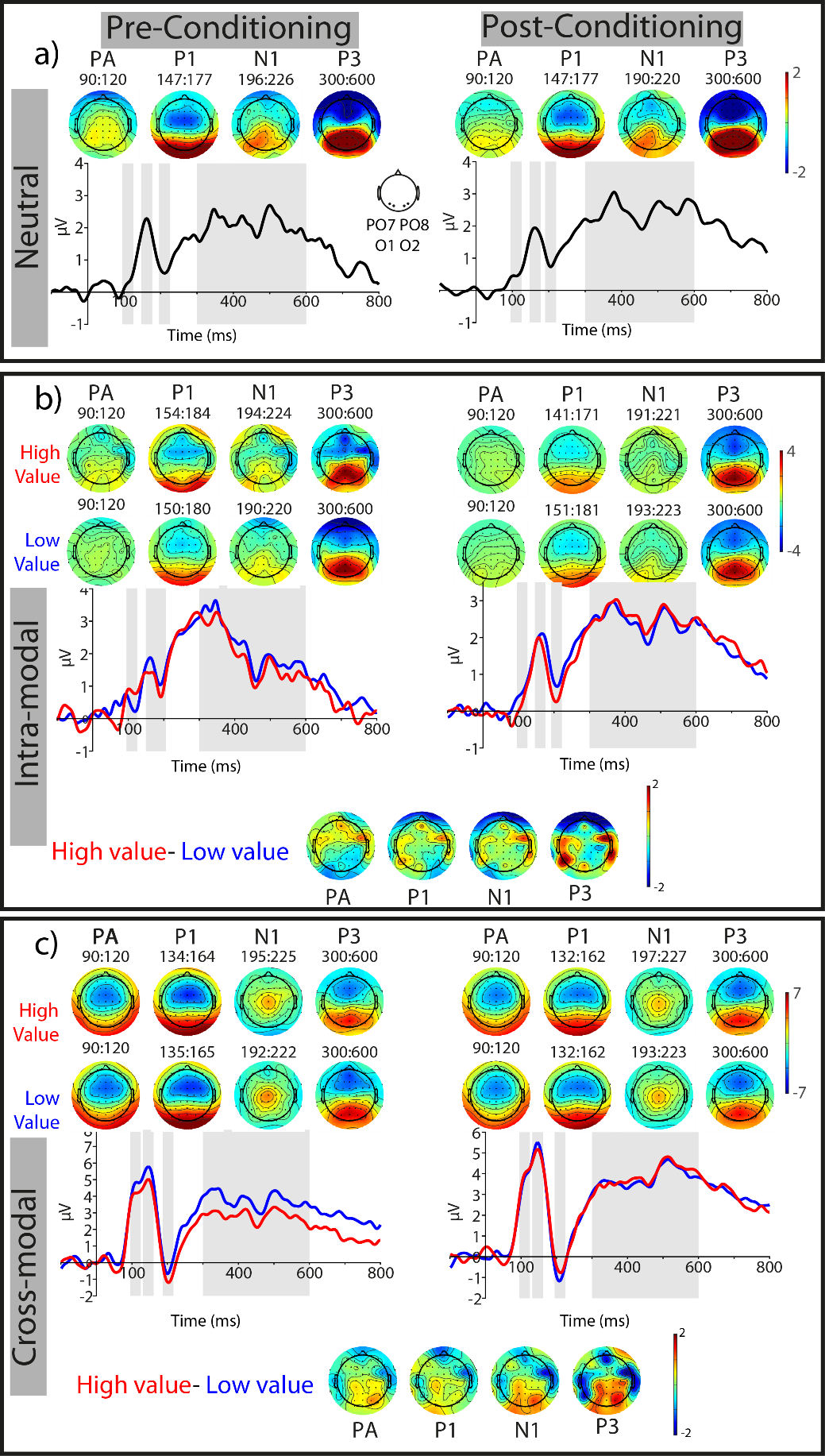
**

**S6 Figure. ERP results of the posterior ROI when only correct trials were included, see also Figure 4 in the main text.**
