# Supplementary material for: Differential effects of intra-modal and cross-modal reward value on perception: ERP evidence
Source: PLoS One. 2023 Jun 30;18(6):e0287900. doi: 10.1371/journal.pone.0287900 (PMC10313067; doi:10.1371/journal.pone.0287900)
Supplement: S7 Fig — (DOCX) [file pone.0287900.s008.docx]

**
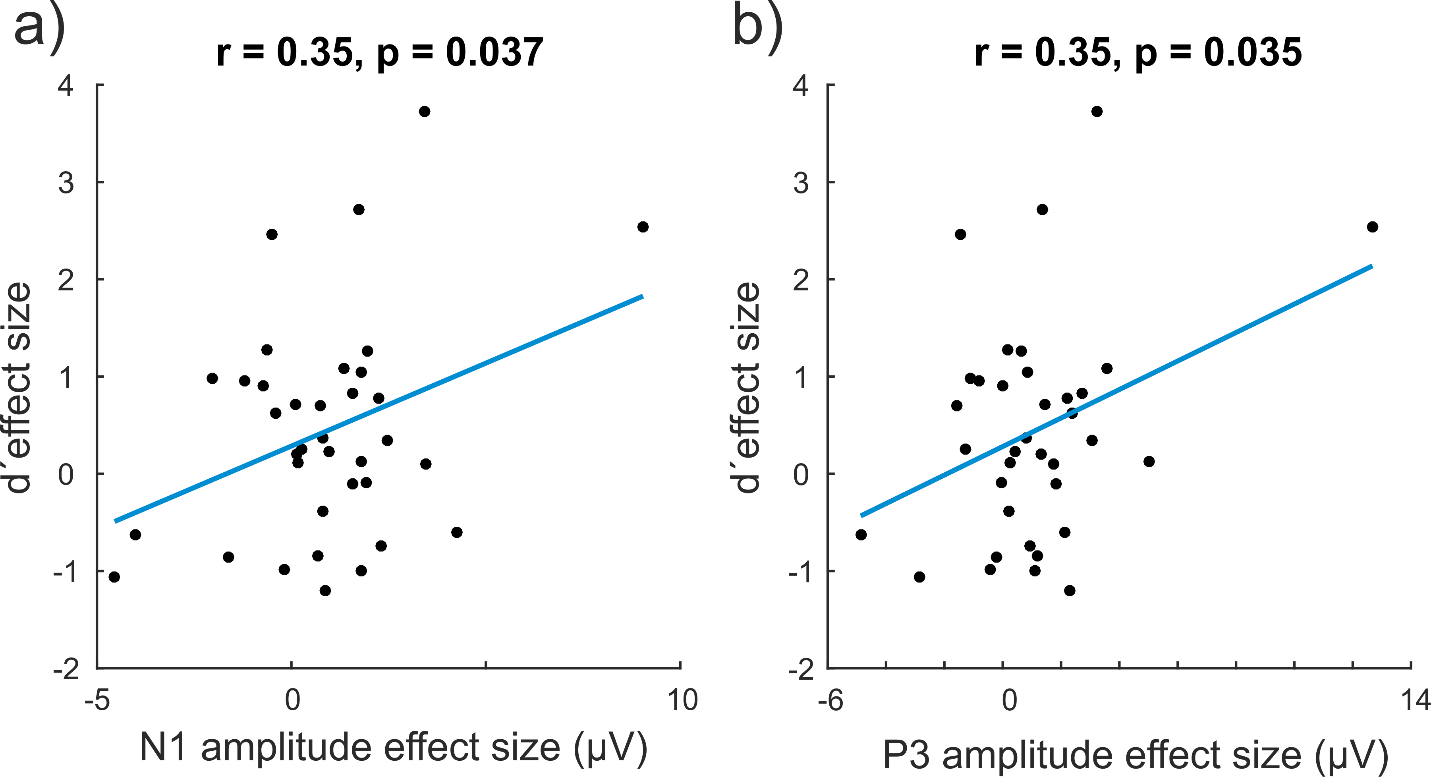
**

**S7 Figure. Correlation between electrophysiological and behavioral effects of reward value. a)** Correlation between reward modulation of contralateral N1 amplitude and d’ in cross-modal condition. Effect size corresponds to the difference of high- and low-value conditions corrected for their difference during pre-conditioning. **b)** Same as **a** for the contralateral P3 amplitude.
