# Supplementary material for: Differential effects of intra-modal and cross-modal reward value on perception: ERP evidence
Source: PLoS One. 2023 Jun 30;18(6):e0287900. doi: 10.1371/journal.pone.0287900 (PMC10313067; doi:10.1371/journal.pone.0287900)
Supplement: Attachment — Submitted filename: RebuttalLetter_secondRevision.docx [file pone.0287900.s010.docx]

# Summary of the decision letter and our response

Comments to the Author

1. If the authors have adequately addressed your comments raised in a previous round of review and you feel that this manuscript is now acceptable for publication, you may indicate that here to bypass the “Comments to the Author” section, enter your conflict of interest statement in the “Confidential to Editor” section, and submit your "Accept" recommendation.

Reviewer #2: All comments have been addressed

Reviewer #3: (No Response)

2. Is the manuscript technically sound, and do the data support the conclusions?

The manuscript must describe a technically sound piece of scientific research with data that supports the conclusions. Experiments must have been conducted rigorously, with appropriate controls, replication, and sample sizes. The conclusions must be drawn appropriately based on the data presented.

Reviewer #2: Yes

Reviewer #3: Yes

3. Has the statistical analysis been performed appropriately and rigorously?

Reviewer #2: Yes

Reviewer #3: Yes

4. Have the authors made all data underlying the findings in their manuscript fully available?

The PLOS Data policy requires authors to make all data underlying the findings described in their manuscript fully available without restriction, with rare exception (please refer to the Data Availability Statement in the manuscript PDF file). The data should be provided as part of the manuscript or its supporting information, or deposited to a public repository. For example, in addition to summary statistics, the data points behind means, medians and variance measures should be available. If there are restrictions on publicly sharing data—e.g. participant privacy or use of data from a third party—those must be specified.

Reviewer #2: Yes

Reviewer #3: Yes

5. Is the manuscript presented in an intelligible fashion and written in standard English?

PLOS ONE does not copyedit accepted manuscripts, so the language in submitted articles must be clear, correct, and unambiguous. Any typographical or grammatical errors should be corrected at revision, so please note any specific errors here.

Reviewer #2: Yes

Reviewer #3: Yes

We thank the editor and the reviewers for their positive evaluation of our work and for their insightful suggestions. In our revisions, we have carefully implemented all reviewers’ suggestions, which have made the manuscript much stronger than the original version. We hope that we have satisfactorily addressed all reviewers’ comments and our manuscript can be now accepted for publication in Plos One.

# Point-by-point response

## Comments of Reviewer 2

Reviewer #2: The authors have fully answered the doubts raised in my previous review. It is very clear now what they meant by "neutral condition" and that the related cue was not associated with any reward. The meanings of the various acronyms have also been clarified in an appropriate manner and also the statistical significance of the various results is now much more readable and understandable.

We thank the reviewer for the very insightful suggestions during the first round of revisions which have greatly helped us to improve our manuscript and make it much stronger than the initial submission.

Reviewer #3: This is a very interesting study investigating the effect of intra-modal and cross-modal value on visual perception and its neural correlates combining psychophysics and EEG. I have been involved in the review process after a first round of review, after the authors already addressed several comments. The study investigates an interesting question, using a solid experimental paradigm. The results are interesting and somehow unexpected, with potentially important implications for our understanding of how the sensory brain encodes reward value. The analysis of the data is sound, and the manuscript is overall well written.

Thank you for the positive evaluation of our work and for the extremely helpful suggestions, which we have carefully implemented as outlined below.

I only have some minor comments:

1-Since the main EEG results are based on the difference between pre and post-conditioning traces (which I think is a very intellingent way of analysing the data), I think it might be helpful to show two separate panels (perhaps in figure 5), the average trace of the difference between post and pre conditioning for HI LI and HC and LC conditions. I think that this would help the reader to appreciate better the results, to complete the information provided by the small maps at the bottom of Figure 4b and c.

Thank you for this great suggestion. Since both Figure 4 and 5 already contain many dense panels, we decided to add the difference waves (for HI-LI and HC-LC) and their corresponding topographic distributions to the Supplementary Information (Supplementary Figure 4) and refer to it in the legend of Figure 4 and in the main text (lines 551 and 558).

2- I find the results that, during the conditioning phase, auditory stimuli elicit a stronger P1 component (larger amplitude and shorter latency) than visual stimuli very intriguing. Could the authors discuss this result? How do they interpret it?

Many thanks for pointing this out. Auditory tones have overall shorter processing latencies compared to visual stimuli [1]. However, as the reviwer noted, it is indeed intriguing that in visual cortex, an area that is dedicated to the processing of visual stimuli, the amplitude of P1 responses is larger for auditory compared to visual stimuli. We think that this finding is due to the relative strength of the visual and auditory stimuli that we utilized: visual stimuli in our experiments were small, transparent colored circles (0.44° in diameter), whereas the auditory stimuli were played at a suprathreshold intensity (70 dB) so that participants could detect the tones very easily. In fact, a different approach would be to equalize the perceived intensity of the auditory and visual stimuli by measuring participants’ detection threshold in a 2AFC paradigm for both stimulus types, similar to previous psychophsical studies [2]. However, in our experience (unplublished data), participants can easily detect auditory stimuli even at very low intensities and therefore using an equalization method for visual and auditory stimuli may yield extremely low intensities for visual stimuli which would make the discrimination between them difficult. Since we measure response latencies and amplitudes of P1 and N1 for each modality separately for all phases of the experiment and correct the data of each condition relative to its pre-conditioing counterpart during the test phase, we do not believe that the pattern of results we report in the test phase is affected by the difference in visual and auditory reward cues intensity. This is however a possibility that could be explored in future studies. We have now added these explanations to line 738-739 of the revised manuscript:

“*In this vein, our results also indicated that the perceived intensity of visual and auditory stimuli might have been different as both P1 and N1 components were stronger for auditory compared to visual stimuli during the conditioning phase. Since we quantified the ERP measures separately for each sensory modality, calculated the reward effects against stimuli from the same modality, and corrected for pre-conditioning biases, it is unlikely that our reported results are due to the differences in perceived intensity of auditory and visual stimuli. We note however that using an adaptive method to equalize the perceived intensity of auditory and visual stimuli would be an interesting addition to future studies.*”

3- During the conditioning phase, is there a difference between the first and the second half of the trials? If participants learn the association between a particular stimulus and either high or low reward, a difference between the two migh emerge later on during the experimental session.

This is a great point. In order to test whether our results during the conditioning (reaction times, P3 amplitude and N1 latencies) are affected by the learning of reward associations which evolves in time, we repeated each analysis by including the phase of learning (i.e., first or second half of conditioning) as a factor. We did not observe a significant interaction between any of the reported effects and the phase of conditioning (first versus second half). These results (below) are now presented in lines 448-457 of the revised manuscript:

“*Since learning of reward associations may take time and behavioral and ERP effects of reward could only arise after associative learning has been completed, we wondered whether our reported results differed between the first and the second half of the conditioning. To test this possibility, we divided the trials for each condition to two halves and entered an extra factor, i.e., phase (first or second half of conditioning) in all our ANOVAs. We found similar effect sizes in each case (the interaction between reward and modality for RT: F(1,35) = 7.70, p = 0.009, ηp2= 0.18 and for N1 latency: F(1,35) = 7.26, p = 0.011, ηp2= 0.172, and the main effect of reward on P3 amplitude: F(1,35) = 4.27, p = 0.046, ηp2 = 0.109), but we did not observe a significant interaction with the phase (all ps>0.1). Therefore, our reported results did not show a dependence on the phase of conditioning, likely because full learning of reward associations was achieved very fast.”*

4-Figure 3e-f and Figure 5c: individual subjects data could be overlayed on top of the bar plots so to better appreciate the interindividual variability of the effect.

Thanks for this suggestion. We have now added the individual data to both figures (3e-f and 5c).

line 124: Figure 4d actually refers to Figure 2b, please correct the typo.

Thank you for brining this error to our attention (it was in line 424). We have now corrected the typo (from Figure 4d to Figure 2b).

1.	King AJ. Multisensory Integration: Strategies for Synchronization. Curr Biol. 2005;15: R339–R341. doi:https://doi.org/10.1016/j.cub.2005.04.022

2.	Ragot R, Cave C, Fano M. Reciprocal effects of visual and auditory stimuli in a spatial compatibility situation. Bull Psychon Soc. 1988;26: 350–352. doi:10.3758/BF03337679
